# P-164. Global Childhood Diarrhea Prevalence and Its Determinants: A Systematic Meta-Analytic Assessment, 1985–2024

**DOI:** 10.1093/ofid/ofaf695.388

**Published:** 2026-01-11

**Authors:** Zahir Tag, Hadeel Alashwal, Hiam Chemaitelly, Laith Abu-Raddad

**Affiliations:** Weill Cornell Medicine - Qatar, Doha, Ad Dawhah, Qatar; Weill Cornell Medicine - Qatar, Doha, Ad Dawhah, Qatar; Weill Cornell Medicine - Qatar, Doha, Ad Dawhah, Qatar; Weill Cornell Medicine - Qatar, Doha, Ad Dawhah, Qatar

## Abstract

**Background:**

Childhood diarrhea continues to be a major contributor to morbidity and mortality among children under five years of age. This study provides an analysis of global childhood diarrhea prevalence, drawing on standardized, nationally representative survey data collected over a four-decade period from 1985 to 2024.

**Methods:**

A systematic review of Demographic and Health Surveys and Multiple Indicator Cluster Surveys conducted up to October 30, 2024, was undertaken, with findings reported in accordance with PRISMA guidelines. Random-effects meta-analyses were used to estimate pooled mean prevalence, while meta-regression analyses were conducted to examine associations and assess temporal trends. Factor analysis was employed to construct a Socioeconomic and Child Nutrition Index, integrating socioeconomic, water, sanitation, hygiene, and nutrition indicators.

**Results:**

The analysis identified 593 relevant studies, estimating a global pooled mean childhood diarrhea prevalence of 14.4% (95% CI: 13.8–15.0%) across all regions and time periods. Prevalence declined at a rate of 1% per year, falling from 22.2% in 1985–1989 to 10.9% in 2020–2024, with consistent declines observed in all regions except the Eastern Mediterranean Region. Prevalence was highest in the African Region and lowest in the European Region. Higher prevalence was observed in countries with larger household sizes, longer water collection times, and higher rates of underweight, stunting, and wasting. Conversely, lower prevalence was found in countries with greater urbanization, higher maternal education, population density, Human Development Index, income per capita, and access to improved water sources and sanitation facilities. A higher Socioeconomic and Child Nutrition Index was strongly and consistently associated with lower diarrhea prevalence, demonstrating a dose–response relationship.

**Conclusion:**

Childhood diarrhea prevalence has declined over recent decades, reflecting the combined impact of public health interventions and broader structural improvements. These findings underscore the need for an integrated approach that pairs targeted health strategies with sustained socioeconomic development to address upstream determinants and support long-term progress in child health.

**Disclosures:**

All Authors: No reported disclosures

